# The Utility of Olfactory Testing to Discriminate Parkinson's Disease From Diagnostic Mimics: A Systematic Review and Meta‐Analysis

**DOI:** 10.1111/ene.70473

**Published:** 2025-12-30

**Authors:** Christoph Theyer, Johannes Kirchmair, Frank Jagusch, Simon Leiter, Atbin Djamshidian, Beatrice Heim, Corinne Horlings, Alessandra Fanciulli, Werner Poewe, Klaus Seppi, Philipp Mahlknecht, Florian Krismer

**Affiliations:** ^1^ Department of Neurology Innsbruck Medical University Innsbruck Austria; ^2^ Department of Neurology Kufstein District Hospital Kufstein Austria

**Keywords:** essential tremor, multiple system atrophy, olfaction, Parkinson disease, progressive Supranuclear palsy

## Abstract

**Background:**

Hyposmia is present in most patients with Parkinson's disease (PD), whereas olfaction is usually preserved in its diagnostic mimics. To address the limited evidence from smaller studies, we conducted a meta‐analysis on the diagnostic accuracy of olfactory testing in differentiating PD from clinical look‐alikes.

**Methods:**

A systematic search was performed in PubMed and Web of Science according to the PRISMA guidelines. Studies describing results of validated smell tests in PD patients and at least one differential diagnosis were included. The risk of bias and applicability was assessed with the QUADAS‐2 tool. For data synthesis, a hierarchical regression model was employed.

**Results:**

Of 787 publications, 23 studies describing 1957 PD patients, 462 patients with atypical parkinsonian disorders, 239 patients with essential tremor, and 43 patients with secondary parkinsonism were included. The meta‐analysis demonstrated a sensitivity of 79% (95% confidence interval [CI]: 72%–84%) and a specificity of 81% (95% CI: 73%–86%) for olfactory dysfunction to differentiate PD from all other disorders combined. Additional analyses showed consistent sensitivities across sub‐analyses, with lowest specificities for the distinction from progressive supranuclear palsy, 64% (95% CI: 55%–72%), and highest from essential tremor, 92% (95% CI: 84%–96%).

**Conclusion:**

Our findings indicate that olfactory testing shows moderate to good diagnostic accuracy in differentiating PD from its main differential diagnoses. While results of olfactory testing alone are insufficient for a definite distinction of PD from non‐PD parkinsonism, it represents an easy‐to‐use and inexpensive test that may be used in combination with other diagnostic tools.

## 1 Introduction

Misclassification of patients with Parkinson's disease (PD) in daily clinical practice is still common due to overlapping clinical presentations with other types of parkinsonism or non‐PD tremor disorders. Clinicopathological studies have found misclassification rates ranging from 17% to 26% depending on the clinical setting [1, 2] with initial distinction of patients with atypical parkinsonian disorders (APD), such as multiple system atrophy (MSA) and progressive supranuclear palsy (PSP), being most challenging. Difficulties may also arise in distinguishing secondary forms of parkinsonism as well as related tremor syndromes, like essential tremor (ET) or dystonic tremor from PD [1, 2, 3].

Hyposmia is present in most patients with PD and listed as a feature supporting a diagnosis of PD in the MDS clinical diagnostic criteria [3], while olfactory function is usually preserved in non‐PD parkinsonism [4, 5, 6, 7, 8, 9, 10]. Olfactory function can be assessed by a variety of inexpensive and easily applicable tests [11], some of which exist in culturally adapted versions for use in diverse populations worldwide [12, 13]. Since the prevalence of olfactory dysfunction (OD) in the overall population increases with age and is higher in males than females, age‐ and sex‐adjusted cut‐off values are applied in most of the commonly used tests [11, 12, 14]. In addition, different olfactory domains can be tested separately with odor identification tests featuring a higher diagnostic value in identifying PD patients as compared to testing of olfactory discrimination and threshold [11].

Many studies have assessed the usefulness of OD for differentiating PD from related diseases, with most of them reporting satisfactory diagnostic accuracy [5, 15]. However, these studies used different olfactory tests with variable cut‐off values in mostly small samples. In the present work, we sought to perform a systematic review and meta‐analysis on studies that used established and well‐validated smell tests and report the accuracy of OD in distinguishing PD from related diseases.

## 2 Methods

The current work was registered in the International prospective register for systematic reviews (PROSPERO; registration number CRD 42024601899). This systematic review and meta‐analysis was performed according to the PRISMA guidelines [16].

### 2.1 Eligibility Criteria

Articles published in English language in peer‐reviewed journals describing olfactory tests in PD and at least one of its most common differential diagnoses (i.e., MSA, PSP, corticobasal syndrome [CBS], ET, dystonic tremor or secondary parkinsonism) were included. Patients with CBS were included in the PSP group [17]. In addition, included studies had to: (1) contain the name of the smell test and the number of odors, (2) describe the process by which diagnoses were assigned (based on expert clinical examination, clinical diagnostic criteria, or neuropathologic examination), and (3) provide sufficient information to enable calculation of sensitivities and specificities of the smell test for final diagnosis.

### 2.2 Data Sources

A comprehensive search was done on the 16th of October 2024 in MEDLINE/PubMed and Web of Science with articles included until October 2024. Rayyan [18], a web‐tool designed to assist within the screening process, was used for the systematic search. The databases were queried with the following keywords and MeSH terms: (“multiple system atrophy” OR MSA OR “olivopontocerebellar atrophy” OR OPCA OR “striatonigral degeneration” OR SND OR “Shy‐Drager syndrome” OR “progressive supranuclear palsy” OR PSP OR “Richardson*” OR (“multiple system atrophy“[MeSH Terms]) OR (“progressive supranuclear palsy“[MeSH Terms]) OR CBD OR CBS OR corticobasal* OR (“Corticobasal Degeneration“[MeSH Terms]) OR atypical* OR tremor* OR (“Tremor”[Mesh]) OR drug‐induced* OR “drug induced “OR vascular* OR (“Parkinson Disease, Secondary”[Mesh]) AND (odor OR olfact* OR sniff* OR smell* OR anosm* OR hyposm*) AND (parkinson OR (“Parkinson Disease”[Mesh])).

### 2.3 Article Selection and Data Extraction

Duplicates were removed with the help of AI‐assisted Rayyan duplication detection [18]. Two independent reviewers (C.T. and J.K.) screened all articles. After the screening process, both reviewers discussed differences and if inconsistencies occurred, a third, senior reviewer (P.M. or F.K.) decided on further inclusion. Subsequently, full text screening was performed by the same independent reviewers and inclusion and exclusion criteria were applied. Citation searching was done in all included studies, which did not lead to identification of further articles. Data on olfactory performance, smell test information, age, sex, disease duration, age at onset, disease severity and, if available, cognitive performance were tabulated. If optimal cut‐offs for differentiation using a receiver operating characteristic (ROC) curve were given, data on sensitivity and specificity were extracted. Further, if a pre‐defined cut‐off was used and sensitivity and specificity could be either extracted from the text or a graph, the pre‐defined cut‐off described in the same article was used. If neither an optimal cut‐off nor a pre‐defined cut‐off was given and we were able to gather data on olfactory performance, we used well established and validated cut‐offs previously described in other studies [19, 20].

### 2.4 Quality Assessment

A quality and bias assessment using the Quality Assessment of Diagnostic Accuracy Studies 2 (QUADAS‐2) tool was done for all included articles by two independent reviewers (C.T. and F.J.) [21]. In general, the QUADAS‐2 tool is a widely used framework for evaluating the methodological quality of diagnostic accuracy studies, combining both the risk of bias and concerns regarding applicability. The purpose is to systematically assess four different domains (patient selection, index test, reference standard, flow and timing) for potential limitations in the included studies [21]. Results from the quality assessment were compared and if discrepancies occurred a third reviewer (F.K. or P.M.) was involved. QUADAS‐2 worksheets were not changed for this specific research question. The QUADAS‐2 graphics were generated using the web‐based tool Robvis [22]. Robvis is an open‐access software based on R, designed to facilitate the creation of scientific visualizations, including those for QUADAS‐2 quality assessments. Data were uploaded in.csv format, and graphics were produced for the “risk of bias domains” and “concerns regarding applicability”.

### 2.5 Statistical Analysis

Sensitivities and specificities of each study were pooled using a hierarchical regression approach. Sensitivity and specificity rates are presented with 95% confidence intervals (95% CI). Between‐study heterogeneity was assessed by the *I* ^2^ statistic, a parameter that provides a measure of the degree of inconsistency across studies describing the percentage of total variation attributable to heterogeneity rather than chance. Statistical analyses were performed using the STATA commands METANDI and MIDAS (StataCorp 2007, Stata Statistical Software, Release 14.2; StataCorp LP, College Station, TX).

Additionally, a meta‐analysis of “higher‐quality” studies was conducted, using only publications that utilized clinical diagnostic criteria, commonly employed olfactory tests (UPSIT, Sniffin Sticks, B‐SIT, OSIT‐J), and exhibited a maximum of two domains of high risk of bias.

## 3 Results

### 3.1 Search Results

**FIGURE 1:**
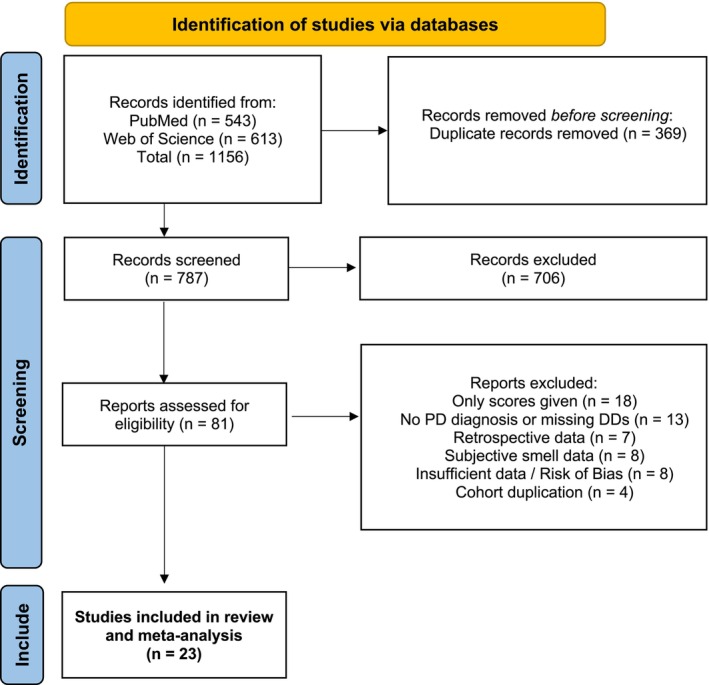
Flow chart for the identification of included studies. DD, differential diagnosis; PD, Parkinson's disease.

After duplication removal a total of 787 publications were identified for abstract screening, of which 81 manuscripts were considered for full text screening (Figure 1). Twenty‐three met inclusion criteria and were included in the meta‐analysis. The characteristics of the 23 included studies are shown in Table 1 and Table S1. Of these, a comparison with MSA patients was performed in 12 studies, 10 studies reported data on PSP patients, 7 studies included ET patients, and 4 studies included patients with secondary parkinsonism. One study investigated OD in subjects without evidence for dopaminergic degeneration (SWEDD). Twenty‐one studies used clinical criteria as diagnostic gold standard, while one study based diagnoses on expert opinion [23], and only one utilized pathologic confirmation for diagnostic accuracy [24]. OD was assessed with six different validated smell tests (Table 1 and Table S1). Most used the UPSIT (*n* = 8) or its abbreviated versions with cultural adaptations (B‐SIT; *n* = 4), the Sniffin Sticks identification test (*n* = 5) or the full TDI (Threshold, Dissemination, Identification)‐Sniffin Sticks (*n* = 2), the Japanese OSIT‐J (*n* = 3), or the Japanese Open Essence test (*n* = 1). Cut‐off values were pre‐defined in 47% of the studies.

**TABLE 1:** Overview of included studies with details on olfactory testing and diagnostic assessments.

Author	Country	Diagnosis	Participants	Olfactory dysfunction % (*n*)	Normal olfaction % (*n*)	Smell test	Cut‐ off	Grouping
Wenning et al. 1995	UK	Parkinson's disease	118	77.1% (91)		UPSIT (40 odor item)	< 25	Optimal
		APD	51		84.3% (43)			
Müller et al. 2002	Germany	Parkinson's disease	37	86.5% (32)		TDI SS (48 odor item)	≤ 20	Calculated
		MSA	8		100.0% (8)			
		PSP	1		100.0% (1)			
		CBS	2		100.0% (2)			
Katzenschlager et al. 2004	UK	Parkinson's disease	18	88.9% (16)		UPSIT (40 odor item)	< 23	Optimal
		Vascular Parkinsonism	14		85.7% (12)			
Shah et al. 2008	UK	Parkinson's disease	64	82.8% (53)		UPSIT (40 odor item)	< 25	Optimal
		ET	59		96.6% (57)			
Goldstein et al. 2009	USA	Parkinson's disease	23	65.2% (15)		UPSIT (40 odor item)	≤ 25	Calculated
		MSA	20		75.0% (15)			
Silveira‐Moriyama et al. 2009/2	UK	Parkinson's disease	191	85.3% (163)		UPSIT (40 odor item)	< 25	Pre‐defined
		MSA	14		42.9% (6)			
Silveira‐Moriyama et al. 2009/1	UK	Parkinson's disease	191	85.3% (163)		UPSIT (40 odor item)	< 25	Pre‐defined
		ET	26		84.6% (22)			
		SWEDD	21		76.2% (16)			
Silveira‐Moriyama et al. 2010	UK	Parkinson's disease	86	65.1% (56)		UPSIT (40 odor item)	< 18	Pre‐defined
		PSP	36		75.0% (27)			
Kikuchi et al. 2011	Japan	Parkinson's disease	42	85.7% (36)		OSIT‐J (12 odor item)	< 9	Optimal
		MSA	42		73.8% (31)			
Suzuki et al. 2011	Japan	Parkinson's disease	94	80.9% (76)		OSIT‐J (12 odor item)	< 8	Optimal
		MSA	15		73.3% (11)			
		PSP	7		71.4% (5)			
Chen et al. 2012	China	Parkinson's disease	37	62.2% (23)		SS (16 odor test)	< 10	Pre‐defined
		ET	26		96.2% (25)			
Borghammer et al. 2014	Denmark	Parkinson's disease	69	84.1% (58)		Adapted SIT (16 odor test)	< 10 females, < 9 males	Pre‐defined
		MSA	4		100.0% (4)			
		PSP	11		54.6% (6)			
		CBS	2		50.0% (1)			
Lopez Hernandez et al. 2015	Spain	Parkinson's disease	30	70.0% (21)		SS (12 odor test)	< 8	Optimal
		ET	21		66.7% (14)			
Sengoku et al. 2015	Japan	Parkinson's disease	13	84.6% (11)		OSIT‐J (12 odor item)	< 8	Optimal
		MSA	11		81.8% (9)			
		PSP	5		60.0% (3)			
		CBS	5		80.0% (4)			
Georgiopoulos et al. 2015*	Sweden	Parkinson's disease	24	70.8% (17)		B‐SIT (12 odor test)	According to Manual	Pre‐defined
		APD	16		68.8% (11)			
		Secondary Parkinsonism	5		60.0% (3)			
Mahlknecht et al. 2016	Austria, Italy	Parkinson's disease	63	84.1% (53)		SS (16 odor test)	≤ 9	Optimal
		MSA	23		87.0% (20)			
		PSP	23		73.9% (17)			
		ET	29		89.7% (26)			
Watanabe et al. 2017	Japan	Parkinson's disease	98	60.2% (59)		Open Essence (12 odor item)	≤ 4	Optimal
		MSA	32		81.3% (26)			
		PSP	17		70.6% (12)			
Wang et al. 2020	China	Parkinson's disease	47	53.2% (25)		SS (12 odor test)	NA	
		ET	42		95.2% (40)			
Elhassanien et al. 2021	Egypt	Parkinson's disease	22	90.9% (20)		TDI SS (48 odor test)	< 25	Optimal
		ET	36		94.4% (34)			
Shill et al. 2021**	USA	Parkinson's disease	76	94.7% (72)		UPSIT (40 odor item)	≤ 25	Optimal
		PSP	24		50.0% (12)			
Postuma et al. 2022	Canada	Parkinson's disease	63	95.2% (60)		B‐SIT (12 odor item)	NA	Pre‐defined
		MSA	4		100.0% (4)			
Dutta et al. 2023	India	Parkinson's disease	40	40.0% (16)		INSIT (10 odor test)	≤ 4	Pre‐defined
		MSA	10		70.0% (7)			
		PSP	20		70.0% (14)			
		Vascular Parkinsonism	10		80.0% (8)			
Pavelka et al. 2023	Luxembourg	Parkinson's disease	702	72.9% (512)		SS (16 odor test)	< 10th percentile	Pre‐defined
		PSP	47		46.8% (22)			
		MSA	12		58.3% (7)			
		Vascular Parkinsonism	14		42.9% (6)			

*Note:* The column “grouping” defines the performed approach to differentiate between olfactory dysfunction and normal olfaction. Pre‐defined = established, previously validated, defined cut‐off before start of the study; optimal = ROC curve calculated cut‐off for optimal sensitivity and specificity; Calculated = no cut‐off was described in the publication therefore a well described, validated cut‐off was applied by the authors. All studies used clinical‐diagnostic criteria for group classification, except for one study relying on expert opinion (*Georgiopoulos et al. 2015) while one study based diagnoses on neuropathological examination (**Shill et al. 2021).

Abbreviations: APD, atypical Parkinsonian Disorders; B‐SIT, Brief Smell Identification Test; CBS, corticobasal syndrome; ET, essential tremor; INSIT, Indian Smell Identification Test; MSA, Multiple System Atrophy; OSIT‐J, Olfactory Smell Identification Test – Japan; PSP, progressive supranuclear palsy; SS, sniffin sticks; TDI, threshold, discrimination, identification; UPSIT, University of Pennsylvania Smell Identification Test.

### 3.2 Quality Assessment

**FIGURE 2:**
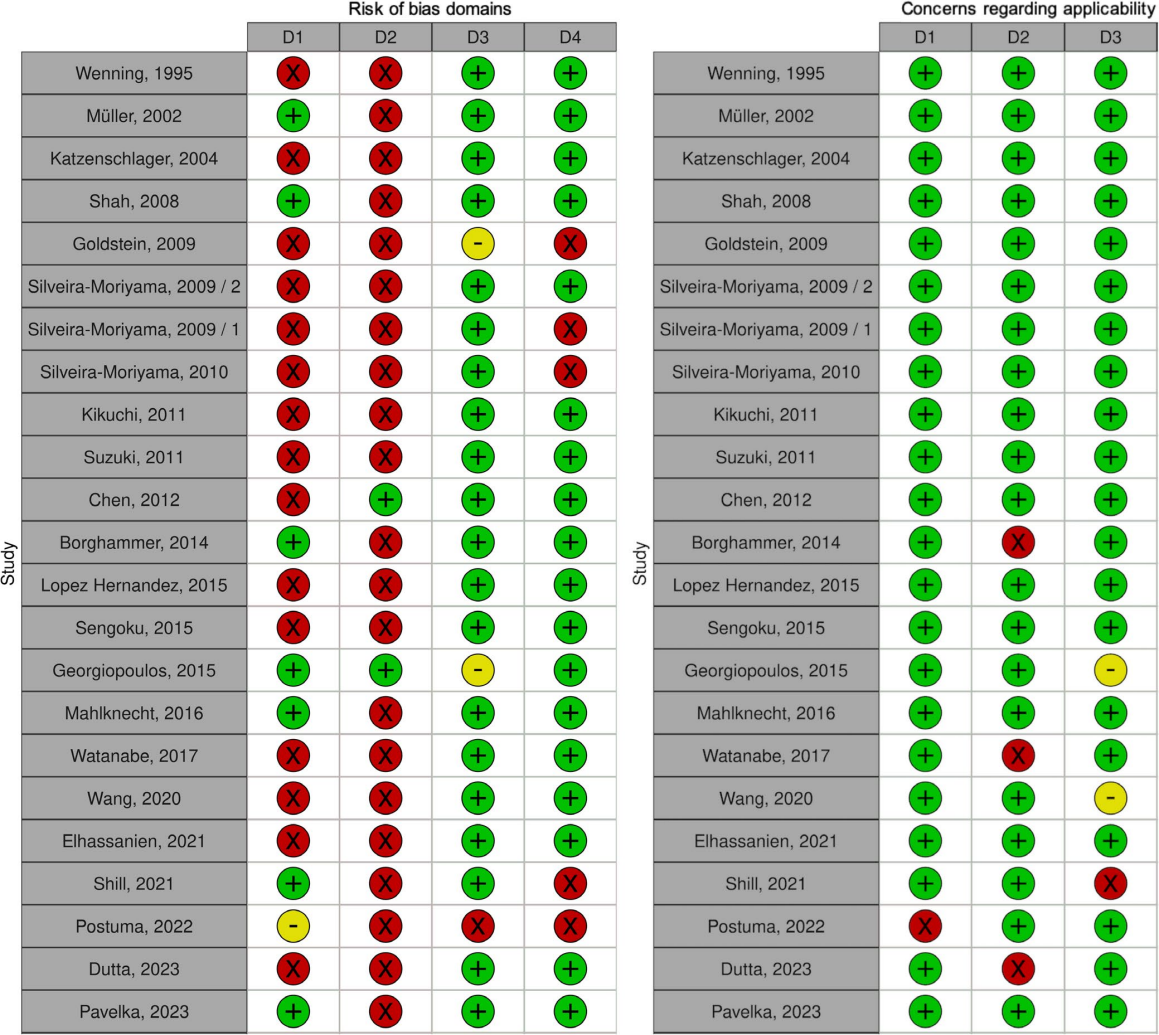
Quality Assessment performed in included studies. The risk of bias domains and concerns regarding applicability were graded in three categories with green displaying low risk/concern, yellow some risk/concerns, and red high risk/concern; Domains: D1 = Patient Selection, D2 = Index Test, D3 = Reference Standard, D4 = Flow & Timing.

The quality assessment showed a high risk of bias in most studies due to two main reasons: (1) lack of random or continuous selection of patients and (2) smell test performed under unblinded conditions (Figure 2). Therefore, the quality assessment revealed a high risk of bias in the patient selection and the index test domains. Moreover, in half of the studies, cut‐offs were not predefined but based on ROC curves for achieving the highest accuracy. Diagnoses were mostly based on published clinical criteria. Applicability concerns were generally low as the aim of our meta‐analysis was to define the value of smell testing as a quick tool for separating PD patients from other diseases, e.g., in an outpatient setting.

### 3.3 Diagnostic Accuracy Using Olfactory Dysfunction

**FIGURE 3:**
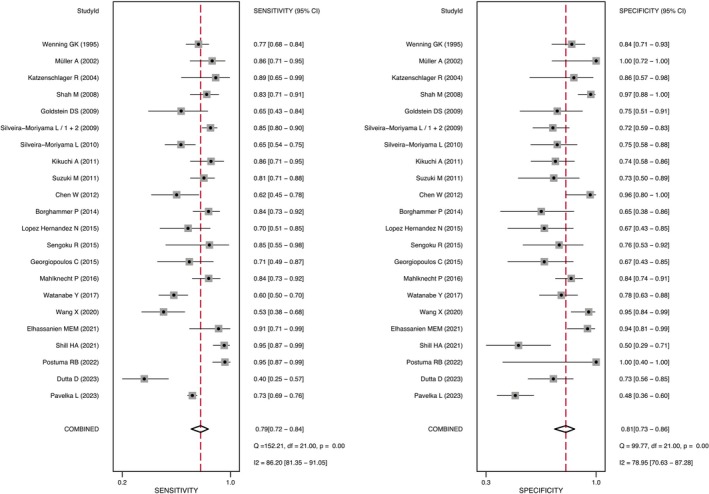
Forrest Plot for all studies combined. The left side represents the sensitivity, where higher values indicate a better ability to correctly identify individuals with Parkinson's disease, while the right side shows the specificity, where higher values indicate a better ability to correctly identify individuals without Parkinson's disease.

All 23 studies were meta‐analyzed to assess the potential of olfactory function testing in distinguishing PD patients from all differential diagnoses combined [5, 6, 7, 8, 15, 23, 24, 25, 26, 27, 28, 29, 30, 31, 32, 33, 34, 35, 36, 37, 38, 39, 40]. Data from 1957 PD, 462 APD, 43 secondary parkinsonism, 239 ET and 21 SWEDD patients were available. OD had a pooled sensitivity of 79% (95% CI: 72%–84%) and a specificity of 81% (95% CI: 73%–86%) for differentiating PD from all differential diagnoses combined (see Figures 2 and 3). Two studies included patients with a disease duration under 3 years and showed a combined sensitivity of 76% (95% CI: 46%–90%) and a specificity of 79% (95% CI: 43%–90%) [29, 34]. A sub‐analysis including only high‐quality studies (i.e., studies that applied widely used smell tests, clinical diagnostic criteria, and that had less than two domains with high risk of bias at QUADAS‐2 quality assessment) exhibited a sensitivity of 81% (95% CI: 76%–85%) and a specificity of 80% (95% CI: 71%–86%; see Figure S1).

#### 3.3.1 PD Versus Atypical Parkinsonian Disorders

**FIGURE 4:**
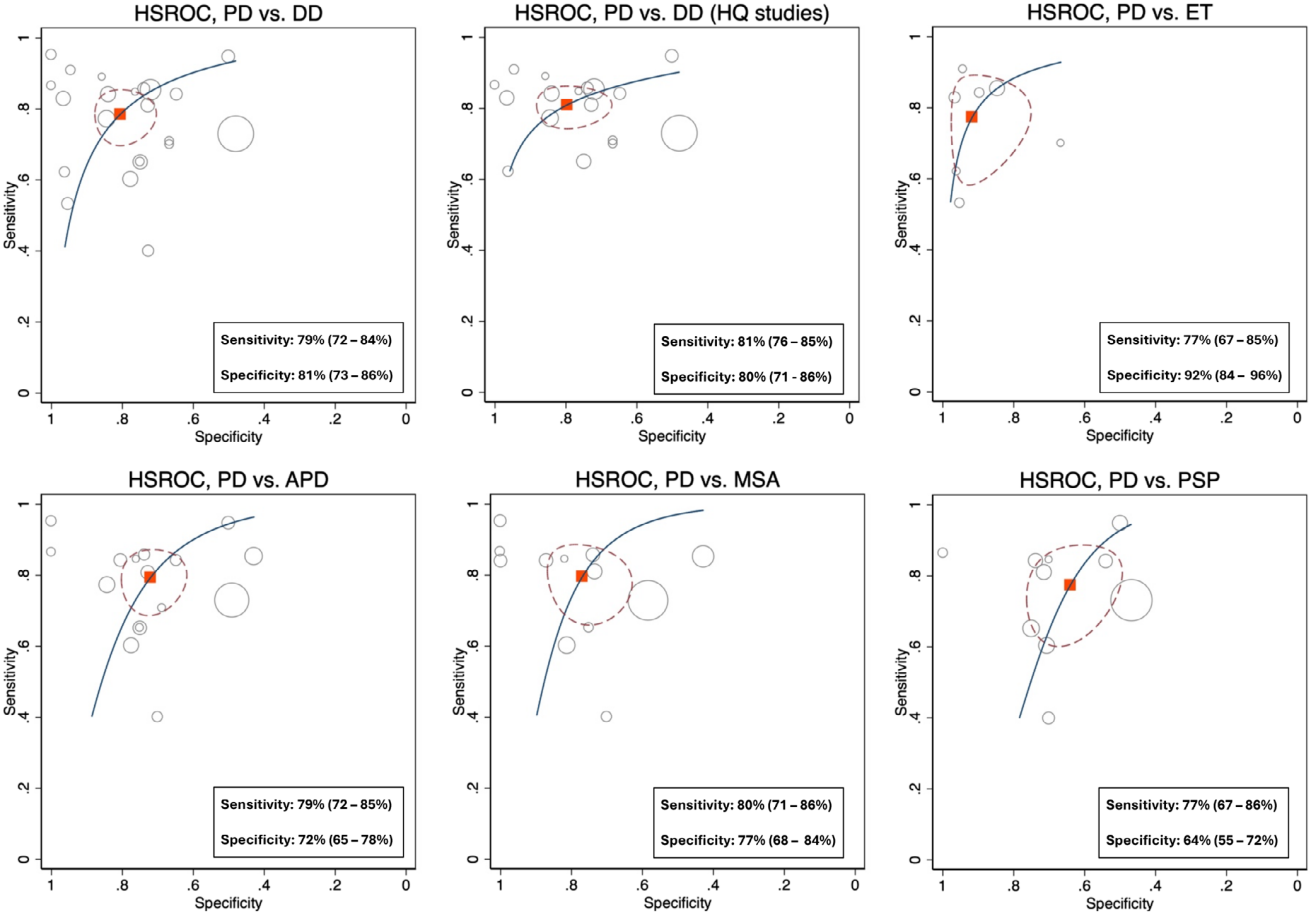
ROC curves of the accuracy between PD and all differential diagnoses combined and separately. Size of the circles represents the number of participants in the studies. Red dotted lines represent 95% CI.

Sixteen studies compared smell testing results in PD (*n* = 1739) versus APDs (*n* = 462). Overall sensitivity and specificity of OD to distinguish between these diseases was 79% (95% CI: 72%–85%) and 72% (95% CI: 65%–78%; see Figure S2). Twelve studies included MSA (195 patients) and 10 studies included PSP (200 patients). Distinction between PD and MSA showed a sensitivity and specificity of 80% (95% CI: 71%–86%) and 77% (95% CI: 68%–84%), respectively (see Figure S3). In comparison, the accuracy of OD to distinguish between PD and PSP showed a sensitivity of 77% (95% CI: 67%–86%) and a specificity of 64% (95% CI: 55%–72%; see Figure 4 and Figure S4).

#### 3.3.2 PD Versus ET

Seven studies compared smell testing performance in patients with PD (*n* = 454) and ET (*n* = 239) [7, 27, 30, 32, 34, 36, 37]. The pooled sensitivity for the distinction of PD from ET was 77% (95% CI: 67%–85%) and the pooled specificity was 92% (95% CI: 84%–96%; see Figure 4 and Figure S5). Notably, five of the included studies reported specificities of 90% or higher, underscoring the potential of olfactory testing as a highly specific tool in distinguishing these two diseases.

#### 3.3.3 PD Versus Secondary Parkinsonism

Secondary parkinsonism was described in four studies. While all of these included vascular parkinsonism, one also evaluated drug‐induced parkinsonism [23, 25, 39, 40]. Across individual studies, accuracy was very heterogeneous with sensitivities ranging from 40% to 89% and specificities from 43% to 86% (see Table 1 and Figure S6).

## 4 Discussion

This meta‐analysis of 23 studies demonstrates a sensitivity of 79% (95% CI, 72%–84%) and a specificity of 81% (95% CI, 73%–86%) of OD for differentiating PD from various differential diagnoses. When evaluating only higher quality studies, the sensitivity slightly increases to 81% (95% CI, 76%–85%) while retaining a similar specificity. Intriguingly, the sensitivity corresponds to the prevalence of OD in PD where previous large studies found hyposmia in approximately 80% of patients [41]. Notably, lower sensitivities were found in Chinese and Indian populations, potentially implying cultural or ethnic differences in olfactory performance in PD patients [30, 36, 39].

With regard to the differentiation of PD from its most important and frequent diagnostic mimics, specificities depended on the assessed differential diagnosis. For the differentiation of PD from MSA, the pooled specificity was 77% (95% CI, 68%–84%), indicating that more than 20% of patients clinically diagnosed with MSA may also feature hyposmia. At first glance this may seem in contrast with the fact that the MDS diagnostic criteria for MSA lists unexplained anosmia as an exclusion criterion for MSA [10]. However, in the present paper, we also included studies that investigated the discriminative power of hyposmia (as opposed to the more strict anosmia definition) and combined hyposmia and anosmia under the label OD, possibly resulting in lower specificity rates. Additionally, the variably defined cut‐off values for the same test across different studies may have also had an impact on the diagnostic yield. Tauopathies, including PSP (and CBS), have been more frequently associated with OD than MSA [4, 31, 35]. In our study, the specificity of OD in differentiating PD from tauopathies was 64% (95% CI, 55%–72%). A neuropathologically confirmed case series suggests that presence of co‐pathologies, potential clinical misdiagnosis, and earlier cognitive difficulties contributes to the frequent presence of OD in PSP [24].

The diagnostic utility of olfactory testing in PD and APDs also depends on the clinical setting due to differences in disease prevalence that markedly influence predictive values of diagnostic tests. At general neurologists' offices, the proportion of PD among patients presenting with parkinsonism is high at approximately 90%. Subsequently, the positive predictive value of OD would exceed 95%, underscoring the test's effectiveness in confirming a PD diagnosis in this setting. In contrast, in specialized outpatient clinics of tertiary referral centers, where the relative prevalence of PD among patients with parkinsonism may be much lower at around 70% (due to a higher proportion of APD), the positive predictive value would decrease to 87% [4, 42, 43]. Additionally, two publications reported on patients in early disease stages (disease duration under 3 years) and diagnostic accuracy remained relatively unchanged [29, 34]. This adds to the potential of olfactory testing to differentiate PD and APDs as a diagnostic tool in early disease phases, when diagnosis may still be uncertain.

In studies evaluating OD in ET, the majority of case–control studies found no difference in olfactory function between ET patients and healthy controls [44]. In this meta‐analysis, the specificity of OD for the differentiation of PD from ET patients was high at 92% (95% CI, 84%–96%), with all but one study observing a specificity above 80% [32], suggesting that olfactory testing can differentiate patients with PD (and PD tremor) from ET with high accuracy.

The differential diagnosis of secondary, particularly drug‐induced parkinsonism and vascular parkinsonism, is highly relevant in clinical practice [45, 46]. Due to limited availability of studies on drug‐induced parkinsonism and OD, our meta‐analysis focused on vascular pathologies. It revealed inconsistent findings with specificities of OD to distinguish PD from secondary parkinsonism ranging from 43% to 86%. Previous publications proposed that the unmasking of underlying PD pathology by secondary parkinsonism might account for these inconsistencies and persistent OD in what appears to be secondary parkinsonism [47, 48].

A previous meta‐analysis looked at differences in scores on olfactory testing between PD, APD and tremor syndromes and, as expected, found that smell test performance was lowest in PD compared with its differential diagnoses. However, this study did not report associated diagnostic accuracies [9]. One of the most challenging aspects in this area of research is the number of different smell tests applied, different cut‐offs of commonly used smell tests, and different approaches used to define cut‐off values. The 23 studies included in this analysis investigated results from 13 different countries and from six different smell tests. Of the eight studies that employed the UPSIT, three distinct cutoff values were utilized. All studies that applied pre‐defined values employed the threshold for severe hyposmia or anosmia as defined by Doty et al. [8, 26, 27, 49], prior to the recent publication of age‐ and sex‐adjusted UPSIT percentiles in large datasets that were not used by any of the included studies [14]. One study employed a ROC analysis to identify the optimal diagnostic threshold for the UPSIT [25]. While this of course yields the tradeoff for highest overall diagnostic accuracy, it also poses potential risk of bias in the study's design by overfitting the diagnostic accuracy that could affect the generalizability of the results.

Additionally, the wide variation in the number of odors per test, ranging from 10 to 48, poses several challenges. First, it affects the comparability of results across studies, as tests with fewer odors may lack sensitivity and fail to capture subtle olfactory impairments, while those with a higher number of odors may yield more precise results but could also increase participant fatigue and introduce variability in responses [12]. Despite these challenges, we included studies with varying numbers of odors to capture a broader range of smell tests and different approaches. This inclusive strategy allowed us to evaluate the effectiveness of different test formats combined.

There are limitations that must be considered when interpreting the findings of our meta‐analyses. Firstly, the methodological weaknesses of the included studies indicated a high risk of bias particularly in the domains of patient selection and index test. This is mainly associated with the unblinded study designs, the lack of predefined cut‐off values for diagnostic tests and the difficulties in recruiting continuous or random samples and may have led to an overestimation of diagnostic accuracy. However, pre‐selected patients are commonly described in diagnostic meta‐analysis tending to generally increase the sensitivity and specificity [9, 50]. Only one study used neuropathological confirmation for their diagnoses [24], while the other studies applied state of the art clinical diagnostic criteria [3, 51]. In general, olfactory testing can be performed with a variety of tools. These tests can vary in diagnostic accuracy for differentiation between the diseases, limiting the comparability between the tests. Also, different cut‐off values may be a source for inconsistencies; results of a smell test may define OD in one study, while in another even lower scores may indicate normal olfactory function. The current recommended cut‐off values for the UPSIT and Sniffin Sticks test are age‐ and sex‐adjusted. Only a few studies included in this meta‐analysis used adjusted values [31, 40]. Cultural and educational differences may explain some of the heterogeneity of individual study results. However, these drawbacks are inherent to most diagnostic tests, at least to some extent.

In conclusion, we here corroborate the usefulness of olfactory testing as a bedside diagnostic tool to differentiate PD from differential diagnoses with a moderate to good diagnostic accuracy. Among the differential diagnoses, OD is least common in ET, followed by MSA and PSP. While other diagnostic markers, particularly imaging markers, may offer higher accuracy, olfactory testing has advantages such as higher accessibility as well as cost‐ and time‐effectiveness. However, olfactory testing should not be used as a sole diagnostic tool and should always be interpreted in the context of clinical findings and other diagnostic markers. Further studies using clinicopathological findings and either standardized or optimized olfactory tests are needed to assess the true value of OD for differentiation in this evolving field.

## Author Contributions

**Christoph Theyer:** conceptualization, data curation, investigation, project administration, writing – original draft, writing – review and editing. **Johannes Kirchmair:** conceptualisation, data curation, investigation, visualisation, writing – review and editing. **Frank Jagusch:** investigation, project administration, writing – review and editing. **Simon Leiter:** investigation, project administration, writing – review and editing. **Atbin Djamshidian:** writing – review and editing. **Beatrice Heim:** writing – review and editing. **Corinne Horlings:** writing – review and editing. **Alessandra Fanciulli:** writing – review and editing. **Werner Poewe:** writing – review and editing. **Klaus Seppi:** writing – review and editing. **Philipp Mahlknecht:** conceptualization, data curation, investigation, methodology, supervision, writing – original draft, writing – review and editing. **Florian Krismer:** conceptualization, data curation, formal analysis, investigation, methodology, supervision, writing – original draft, writing – review and editing.

## Funding

This study was supported by funds from the Austrian Science Fund (FWF, grant id FG 2700), C.T. was supported by a grant from the MJFF grant ID MJFF‐022057 and by a grant from the Tiroler Wissenschaftsförderung (grant UNI‐0404/2245), and S.L. is supported by a grant from the MJFF grant ID MJFF‐009277.

## Disclosure

C.T., J.K., F.J., S.L. report no disclosures. A.D. reports honoraria from Novo Nodirsk, Bial, Lilly, Esai, and Roche outside of the submitted work; B.H. reports honoraria from Novartis AG, AbbVie, Bial, and grants from the Austrian Science Fund (FWF) outside of the submitted work; C.G.H. reports no disclosures; A. F. reports royalties from Springer Verlag, speaker fees and honoraria from Bial, Theravance Biopharma, CNSystems, Medtronic, Sanofi, Broadview Venures, Austrian Autonomic Society, International Parkinson Disease and Movement Disorders Society, Elsevier and research grants from the FWF‐Austrian Science Fund, Medical University of Innsbruck, Mission MSA and Dr. Johannes and Hertha Tuba Foundation outside of the submitted work; W.P. reports personal fees from Alterity, AbbVie, AC Immune, Bial, Biogen, Britannia, Lilly, Lundbeck, Neuroderm, Neurocrine, Novartis, Orion Pharma, Roche, Sanofi, Takeda, Teva, UCB and Zambon (consultancy and lecture fees in relation to clinical drug development programmed for PD and MSA) and royalties from Thieme, Wiley Blackwell, Oxford University Press and Cambridge University Press and Grand supports from MJFF, EU PF7 and Horizon 2020; K.S. reports honoraria from the International Parkinson and Movement Disorder Society, grants from the FWF Austran Science Fund, the Michael J. Fox Foundation, and the International Parkinson and Movement Disorder Society, as well as personal fees from Teva, UCB, Lundbeck, AOP Orphan Pharmaceuticals AG, AbbVie, Bial, Roche, and Grunenthal outside of the submitted work; P.M. reports no disclosures; F.K. received personal fees from AbbVie, Bial, Institute de Recherches Internationales Servier, Koneksa, Österreichische Apotheker‐Verlagsgesellschaft, Sanofi, Takeda Pharmaceuticals, and Teva in the past 36 months and his institution has ongoing grant support from the Medical University Innsbruck, Austrian Science Fund (FWF) and the Michael J Fox Foundation, outside of the submitted work.

## Ethics Statement

We confirm that we have read the Journal's position on issues involved in ethical publication and affirm that this work is consistent with those guidelines.

## Conflicts of Interest

The authors declare no conflicts of interest.

## Supporting information


**Table S1:** Additional information from the different studies on origin, sex, age, age at onset, disease duration, as well as PD and cognition specific examinations. Numbers were given in mean ± standard deviation. APD, atypical Parkinsonian disorders; CBD, corticobasal degeneration; ET, essential tremor; MDS‐UPDRS, Movement Disorder Society—Unified Parkinson's Disease Rating Scale; MMSE, mini mental status exam; MOCA, montreal cognitive assessment; MSA, multiple system atrophy; PSP, progressive supranuclear palsy.
**Figure S1:** Forrest plot PD vs. all DD combined in “higher quality studies”.
**Figure S2:** Forrest plot PD vs. APD.
**Figure S3:** Forrest plot PD vs. PSP.
**Figure S4:** Forrest plot PD vs. MSA.
**Figure S5:** Forrest plot PD vs. ET.
**Figure S6:** Forrest plot PD vs. secondary parkinsonism.

## Data Availability

All data related to this article can be made available to investigators on reasonable request to the corresponding authors.
