# Supplementary material for: The Utility of Olfactory Testing to Discriminate Parkinson's Disease From Diagnostic Mimics: A Systematic Review and Meta‐Analysis
Source: Eur J Neurol. 2025 Dec 30;33(1):e70473. doi: 10.1111/ene.70473 (PMC12750508; doi:10.1111/ene.70473)
Supplement: Supplementary file 1 — Table S1: Additional information from the different studies on origin, sex, age, age at onset, disease duration, as well as PD and cognition specific examinations. Numbers were given in mean ± standard deviation. APD, atypical Parkinsonian disorders; CBD, corticobasal degeneration; ET, essential tremor; MDS‐UPDRS, Movement Disorder Society—Unified Parkinson's Disease Rating Scale; MMSE, mini mental status exam; MOCA, montreal cognitive assessment; MSA, multiple system atrophy; PSP, progressive supranuclear palsy. Figure S1: Forrest plot PD vs. all DD combined in “higher quality studies”. Figure S2: Forrest plot PD vs. APD. Figure S3: Forrest plot PD vs. PSP. Figure S4: Forrest plot PD vs. MSA. Figure S5: Forrest plot PD vs. ET. Figure S6: Forrest plot PD vs. secondary parkinsonism. [file ENE-33-e70473-s001.docx]

Supplementary Table 1:

| **Author** | **Country** | **Diagnosis** | **Participants** | **Sex (m/f)** | **Age** | **Age at onset** | **Disease Duration** | **MDS UPDRS III Score** | **Hoehn&Yahr Stadium** | **MMSE Score** | **MOCA Score** |
| --- | --- | --- | --- | --- | --- | --- | --- | --- | --- | --- | --- |
| **Wenning GK et al, 1995** | UK | Parkinson's disease | 118 | 58 / 60 | 59,4 ± 11,6 |  |  |  |  |  |  |
|  |  | APD | 51 |  |  |  |  |  |  |  |  |
| **Müller A et al, 2002** | Germany | Parkinson's disease | 37 |  |  |  |  |  |  |  |  |
|  |  | MSA | 8 |  |  |  |  |  |  |  |  |
|  |  | PSP | 1 |  |  |  |  |  |  |  |  |
|  |  | CBS | 2 |  |  |  |  |  |  |  |  |
| **Katzenschlager R et al, 2004** | UK | Parkinson's disease | 18 |  | 70,6 |  | 9,1 |  |  |  |  |
|  |  | Vascular Parkinsonism | 14 |  | 74,1 |  | 6,6 |  |  |  |  |
| **Shah M, et al, 2008** | UK | Parkinson's disease | 64 | 44 / 20 | 67,2 |  |  |  |  |  |  |
|  |  | ET | 59 | 27 / 32 |  |  |  |  |  |  |  |
| **Goldstein DS et al, 2009** | USA | Parkinson's disease | 23 |  | 70 ± 3 |  |  |  |  |  |  |
|  |  | MSA | 20 |  | 59 ± 2 |  |  |  |  |  |  |
| **Silveira-Moriyama L et al, 2009 / 2** | UK | Parkinson's disease | 191 | 114 / 77 | 65,4 ± 10,7 |  | 10,2 ± 6,2 |  |  |  |  |
|  |  | MSA | 14 | 8 / 6 | 61,3 ± 8,6 |  | 6,5 ± 2,8 |  |  |  |  |
| **Silveira-Moriyama L et al, 2009 / 1** | UK | Parkinson's disease | 191 | 114 / 77 | 65,6 ± 10,7 |  |  |  |  |  |  |
|  |  | ET | 26 | 8 / 18 | 69 ± 9,3 |  |  |  |  |  |  |
|  |  | SWEDD | 21 | 10 / 11 | 65,4 ± 11,4 |  |  |  |  |  |  |
| **Silveira-Moriyama L et al, 2010** | UK | Parkinson's disease | 86 | 83 / 57 | 65,6 ± 11,4 |  | 10,4 ± 6,4 | 27,1 ± 10,3 |  | 29 ± 1,1 |  |
|  |  | PSP | 36 | 20 / 16 | 69,2 ± 6,3 |  | 4,8 ± 2,7 | 27,1 ± 10,9 |  | 25,7 ± 3,2 |  |
| **Kikuchi A et al, 2011** | Japan | Parkinson's disease | 42 | 18 / 24 | 64,6 ± 6,5 |  | 2,7 ± 2,6 |  |  |  |  |
|  |  | MSA | 42 | 24 / 18 | 62,9 ± 9,7 |  | 2,6 ± 2,2 |  |  |  |  |
| **Suzuki M et al, 2011** | Japan | Parkinson's disease | 94 | 47 / 47 | 68,6 ± 9,7 |  | 5,1 ± 4,4 | 36,9 ± 17,2 | 2,3 ± 0,8 | 28,4 ± 1,9 |  |
|  |  | MSA | 15 | 9 / 6 | 67,3 ± 9,2 |  | 2,1 ± 1,7 | 34,8 ± 15,6 | 3 ± 0,9 | 28,5 ± 1,9 |  |
|  |  | PSP | 7 | 4 / 3 | 70,6 ± 9,9 |  | 2,4 ± 2 | 48,2 ± 18,1 | 3,1 ± 0,8 | 28,6 ± 1,5 |  |
| **Chen W et al, 2012** | China | Parkinson's disease | 37 | 26 / 11 | 63,8 ± 8,7 |  | 3,6 ± 3,1 | 17,8 ± 12,3 |  | 27,5 ± 2,2 |  |
|  |  | ET | 26 | 14 / 12 | 59,1 ± 12,3 |  | 12 ± 10,8 |  |  | 27,9 ± 1,4 |  |
| **Borghammer P et al, 2014** | Denmark | Parkinson's disease | 69 | 49 / 20 | 65 ± 10 |  |  |  |  |  |  |
|  |  | MSA | 4 | 2 / 2 | 68 ± 5 |  |  |  |  |  |  |
|  |  | PSP | 11 | 3 / 8 | 68 ± 6 |  |  |  |  |  |  |
|  |  | CBS | 2 | 1 / 1 | 66 ± 1 |  |  |  |  |  |  |
| **Lopez Hernandez N et al, 2015** | Spain | Parkinson's disease | 30 | 22 / 8 | 70 ± 10 |  | 2,1 ± 0,9 |  |  |  |  |
|  |  | ET | 21 | 15 / 6 | 67 ± 10 |  | 6,3 ± 3,2 |  |  |  |  |
| **Sengoku R et al, 2015** | Japan | Parkinson's disease | 13 | 8 / 5 | 69,9 ± 10,1 |  | 4,6 ± 3,6 | 18 (14 - 28) | 3,0 (2 - 3) |  |  |
|  |  | MSA | 11 | 7 / 4 | 69,2 ± 7,1 |  | 3 ± 1,5 | 31 (24 - 36) | 4,0 (3 - 4,5) |  |  |
|  |  | PSP | 5 | 4 / 1 | 67,8 ± 8,2 |  | 3,2 ± 1,6 | 19 (12 - 36) | 3,5 (3 - 4) |  |  |
|  |  | CBS | 5 | 3 / 2 | 75,4 ± 4,3 |  | 1,8 ± 0,4 | 25 (19 - 29) | 3,0 (2 - 4) |  |  |
| **Georgiopoulos C et al, 2015** | Sweden | Parkinson's disease | 24 | 13 / 11 | 70,5 |  | 3,5 ± 11,2 |  |  |  |  |
|  |  | APD | 16 | 9 / 7 | 65,5 |  | 4 |  |  |  |  |
|  |  | Second. Parkinsonism | 5 | 5 / 0 | 77 |  | 4 |  |  |  |  |
| **Mahlknecht P et al, 2016** | Austria, Italy | Parkinson's disease | 63 | 84 / 50 | 68 ± 8,8 |  | 6,2 ± 4,8 | 31,3 ± 15,1 | 2,4 ± 0,9 | 28,8 ± 1,3 |  |
|  |  | MSA | 23 | 11 / 12 | 63,3 ± 8,9 |  | 4,2 ± 3,2 | 43 ± 9,2 | 3,3 ± 0,9 | 27,2 ± 2,4 |  |
|  |  | PSP | 23 | 16 / 7 | 67,2 ± 6,2 |  | 3,1 ± 2 | 30,9 ± 10,4 | 3 ± 0,7 | 27 ± 2,1 |  |
|  |  | ET | 29 | 12 / 17 | 74,5 ± 9,8 |  | 18,7 ± 18 |  |  | 28,6 ± 1,7 |  |
| **Watanabe Y et al, 2017** | Japan | Parkinson's disease | 98 | 46 / 52 | 68,9 ± 8 |  | 4,4 ± 4,6 |  | 2,7 ± 0,9 | 27,1 ± 2,8 |  |
|  |  | MSA | 32 | 15 / 17 | 67,2 ± 7,8 |  | 2,6 ± 2 |  | 3,1 ± 1 | 26 ± 2,8 |  |
|  |  | PSP | 17 | 7 / 10 | 71,6 ± 7,9 |  | 2,2 ± 1,5 |  | 3 ± 0,6 | 26,1 ± 2,8 |  |
| **Wang X et al, 2020** | China | Parkinson's disease | 47 | 24 / 23 | 63,4 ± 7 | 56,5 ± 8,1 | 6,8 ± 4,5 | 38,4 ± 17,7 | 2,8 ± 0,9 | 26 ± 3,1 | 22,2 ± 4,7 |
|  |  | ET | 42 | 23 / 19 | 59,1 ± 10,5 | 41,8 ± 16,6 | 17,4 ± 11,5 |  |  | 22,8 ± 8,8 | 18,7 ± 8,1 |
| **Elhassanien MEM et al, 2021** | Egypt | Parkinson's disease | 22 | 14 / 8 | 57,7 ± 3,5 |  | 2,1 ± 0,6 | 45,5 ± 21,4 |  |  |  |
|  |  | ET | 36 | 20 / 16 | 62,6 ± 4,6 |  | 13,4 ± 4,4 |  |  |  |  |
| **Shill HA et al, 2021** | USA | Parkinson's disease | 76 | 56 / 20 |  |  |  |  |  |  |  |
|  |  | PSP | 24 | 17 / 7 |  |  |  |  |  |  |  |
| **Postuma RB et al, 2022** | Canada | Parkinson's disease | 63 | 44 / 19 | 69,5 ± 7,9 |  |  | 6,7 ± 5,2 |  |  | 24,7 ± 3,3 |
|  |  | MSA | 4 | 2 / 2 | 54,3 ± 7,8 |  |  | 2,5 ± 4,4 |  |  | 27 ± 0 |
| **Dutta D et al, 2023** | India | Parkinson's disease | 40 | 28 / 12 | 55,4 ± 10,1 | 51,3 ± 10,3 | 4,2 ± 4,8 | 25,2 ± 12,4 |  |  | 26,4 ± 2,9 |
|  |  | MSA | 10 | 9 / 1 | 59,9 ± 4,6 | 59 ± 5,1 | 2,5 ± 1,6 | 30,1 ± 9,4 |  |  | 27,5 ± 3 |
|  |  | PSP | 20 | 19 / 1 | 61,4 ± 6,6 | 60 ± 6,7 | 2,4 ± 1,3 | 35,1 ± 17,5 |  |  | 20,4 ± 4 |
|  |  | Vascular Parkinsonism | 10 | 9 / 1 | 60,9 ± 11 | 57,2 ± 9,6 | 4,7 ± 5,9 | 33,2 ± 8,2 |  |  | 19,4 ± 6,3 |
| **Pavelka L et al, 2023** | Luxembourg | Parkinson's disease | 702 | 479 / 241 | 67,3 ± 10,9 | 62,3 ± 11,8 | 4,9 ± 5,2 | 35,2 ± 16,3 | 2,2 ± 0,8 |  | 24,3 ± 4,6 |
|  |  | PSP | 47 | 32 / 19 | 70,4 ± 7,5 | 67,6 ± 8,2 | 2,8 ± 2,6 | 53,3 ± 19,5 | 3,3 ± 1,3 |  | 20 ± 6,3 |
|  |  | MSA | 12 | 5 / 7 | 65,9 ± 12 | 62,3 ± 12,6 | 3,8 ± 2,9 | 53,2 ± 22 | 3,3 ± 1,3 |  | 23,2 ± 5 |
|  |  | Vascular Parkinsonism | 14 | 10 / 4 | 73,8 ± 5,6 | 69,9 ± 6,4 | 3,7 ± 2,8 | 42,1 ± 18,9 | 2,8 ± 1 |  | 20,4 ± 6 |

Additional information from the different studies on origin,sex, age, age at onset, disease duration, as well as PD and cognition specific examinations. Numbers were given in mean ± standard deviation.

Abbreviations: MSA = Multiple System Atrophy; PSP = Progressive Supranuclear Palsy; ET = Essential Tremor; APD = Atypical Parkinsonian Disorders; CBS = Corticobasal Syndrome; MDS-UPDRS = Movement Disorder Society – Unified Parkinson’s Disease Rating Scale; MMSE = Mini Mental Status Exam; MOCA = Montreal Cognitive Assessment;


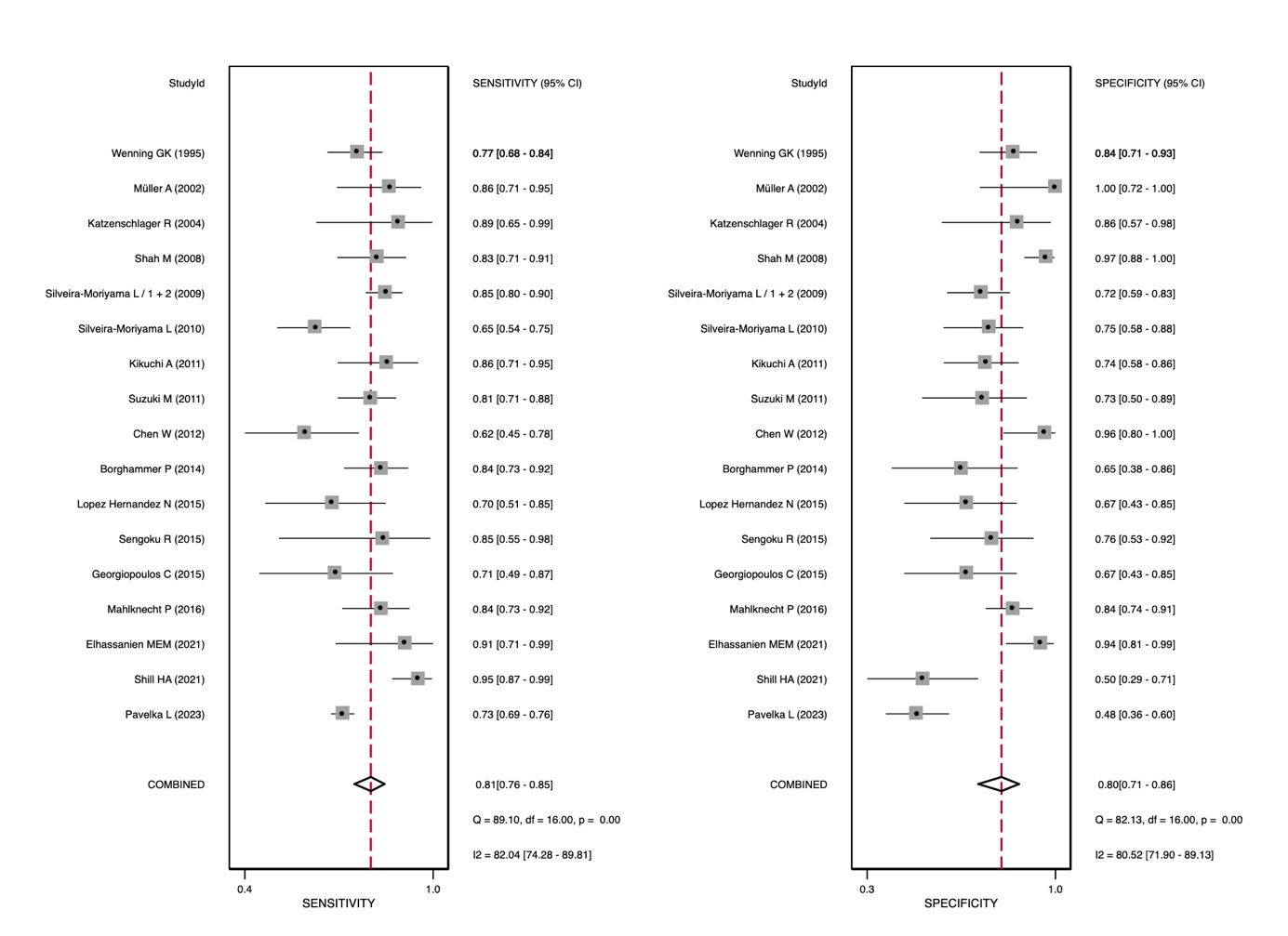
Supplementary Figure 1: Forrest Plot PD vs. all DD combined in “higher quality studies”


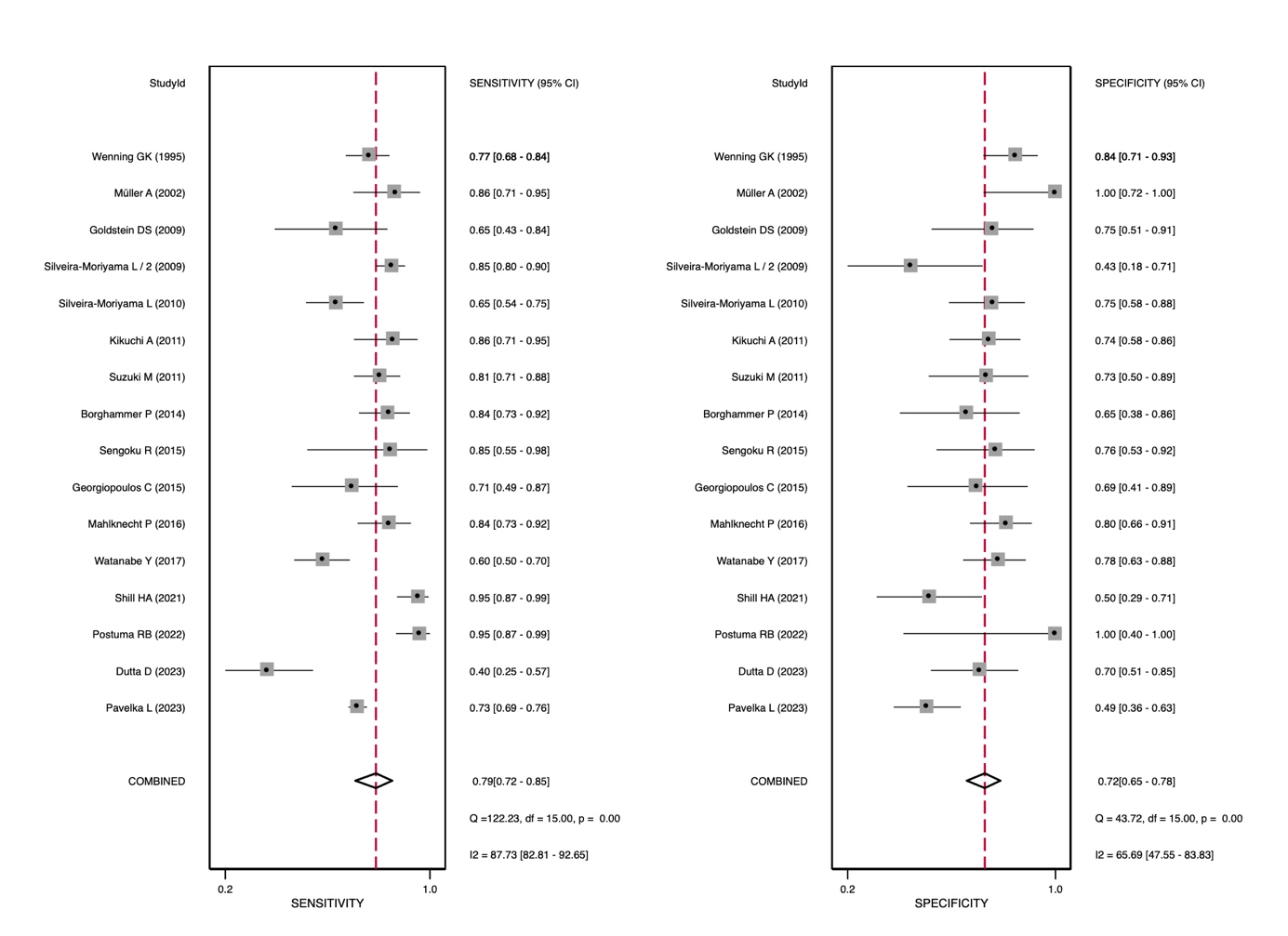
Supplementary Figure 2: Forrest Plot PD vs. APD

Supplementary Figure 3: Forrest Plot PD vs.
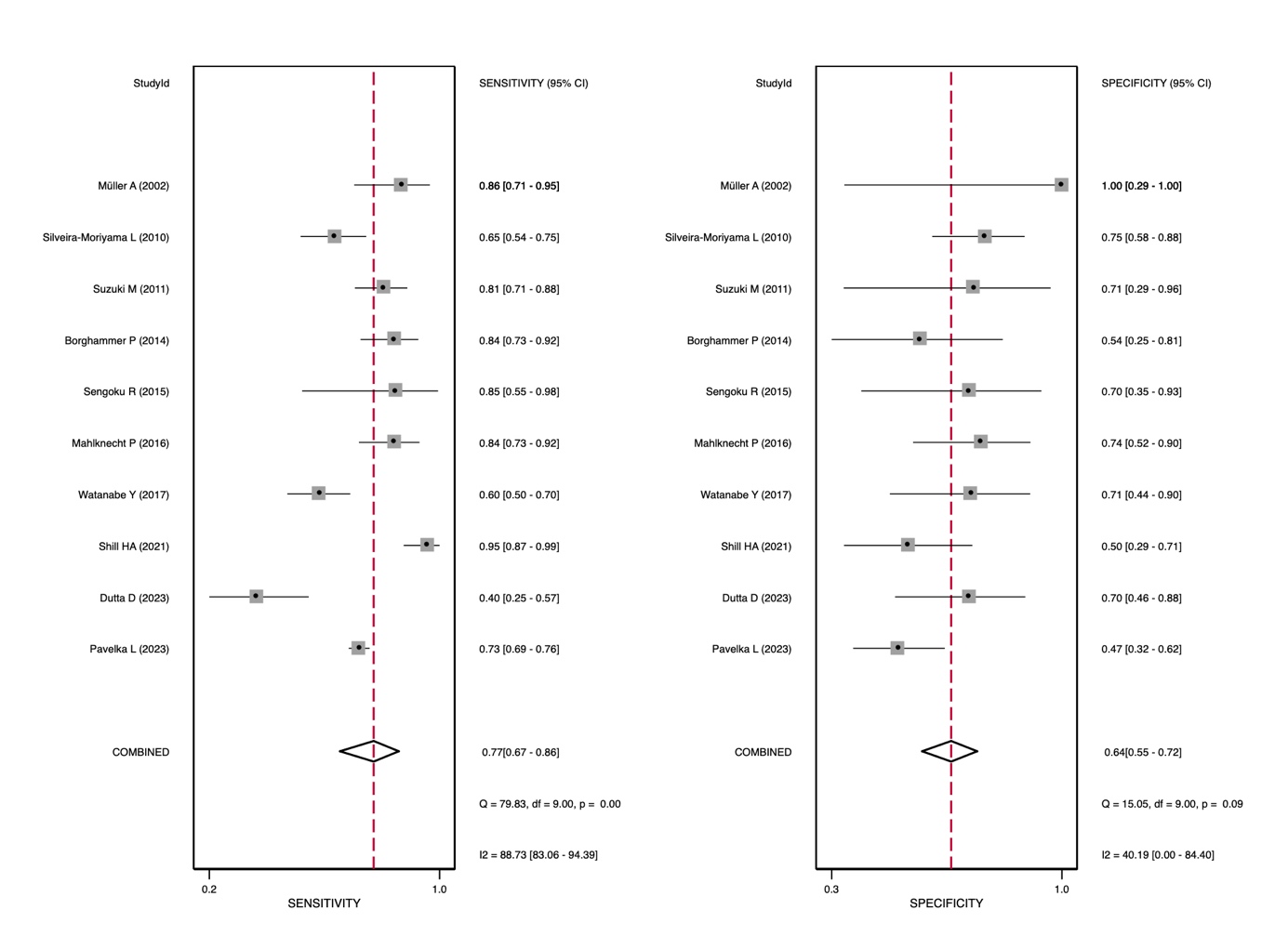
PSP


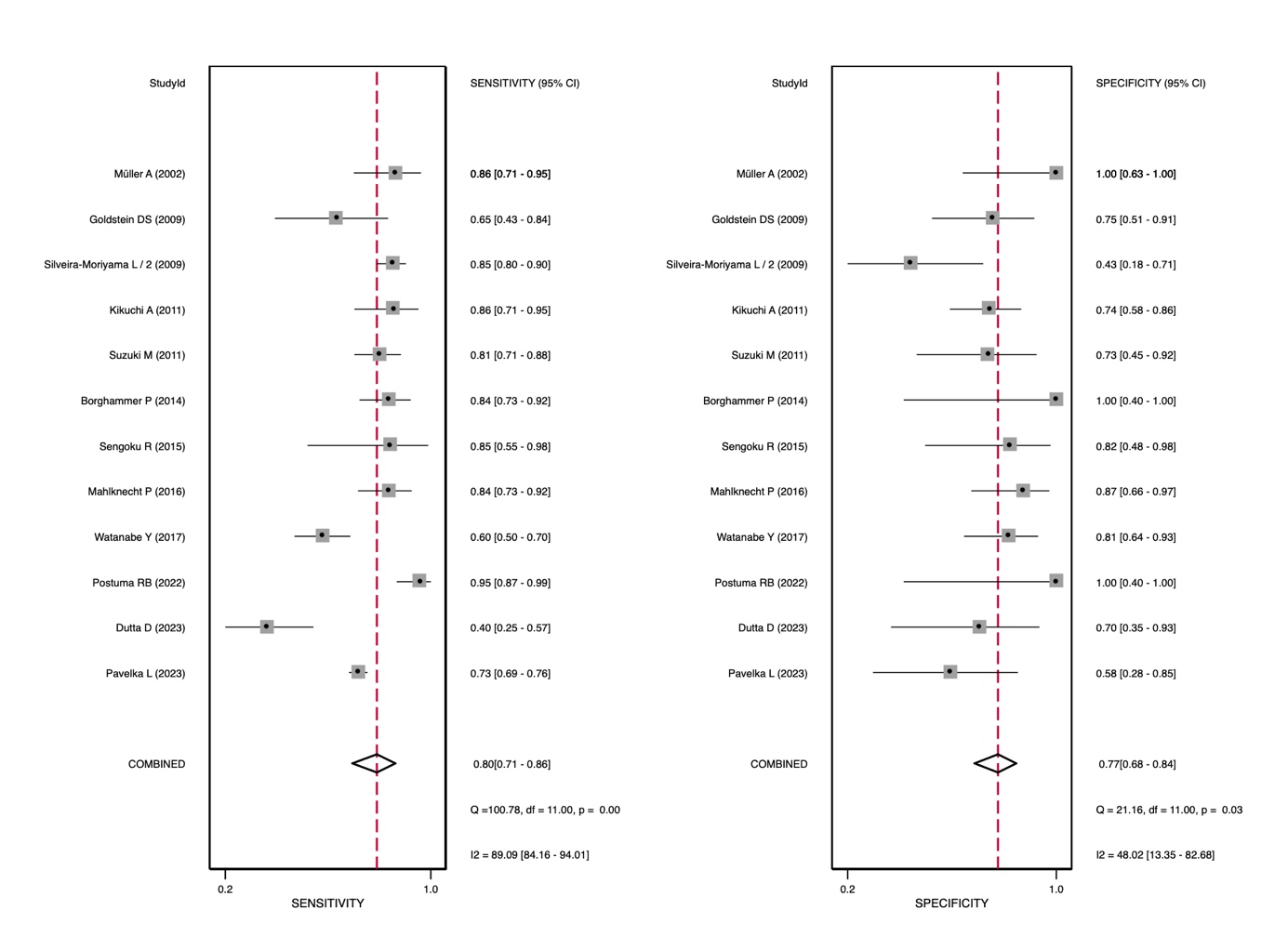
Supplementary Figure 4: Forrest Plot PD vs. MSA


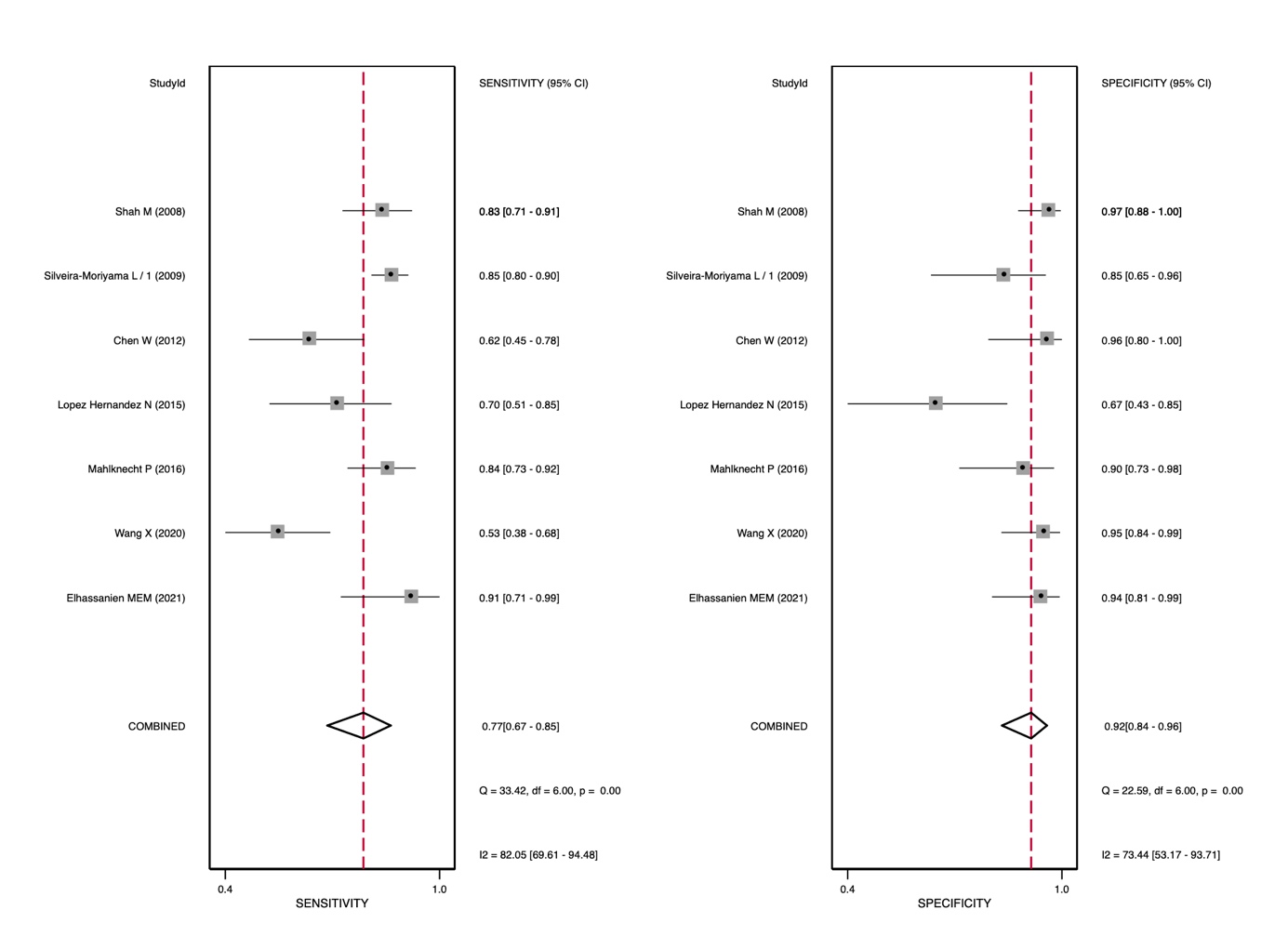
Supplementary Figure 5: Forrest Plot PD vs. ET


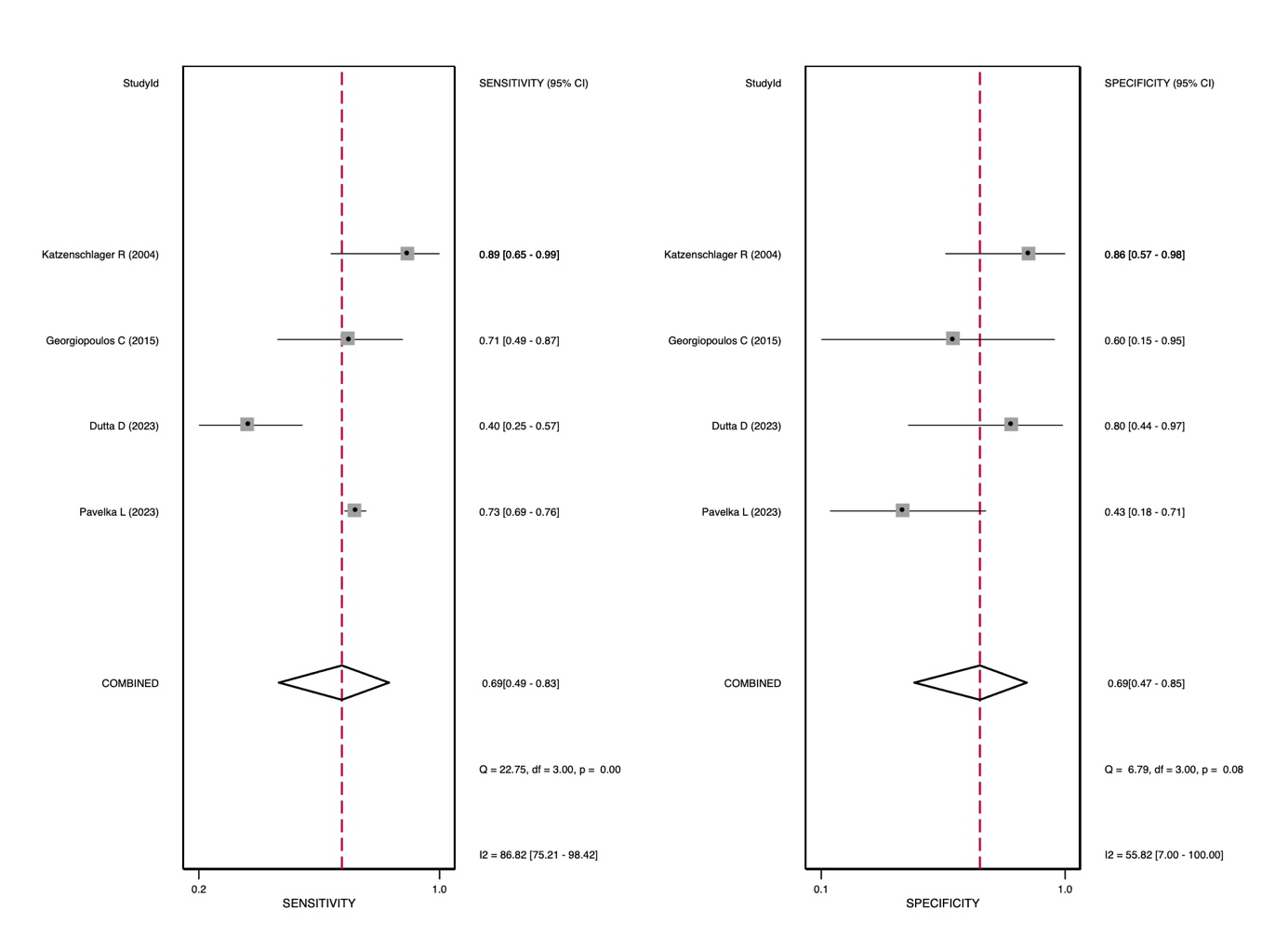
Supplementary Figure 6: Forrest Plot PD vs. secondary parkinsonism
